# Modulation of *Leishmania (L.) amazonensis* Growth in Cultured Mouse Macrophages by Prostaglandins and Platelet Activating Factor

**DOI:** 10.1155/S0962935194000177

**Published:** 1994

**Authors:** M. V. C. Lonardoni, C. L. Barbieri, M. Russo, S. Jancar

**Affiliations:** 1 Department of Biochemistry Universidade Estadual de Maringá Paraná Brazil; 2 Department of Microbiology Immunology and Parasitology Escola Paulista de Medicina São Paulo Brazil; 3 Department of Immunology Instituto de Ciências Biomédicas Universidade de São Paulo Av. Prof. Lineu Prestes 2415 São Paulo CEP 05508-900 Brazil

## Abstract

The role of endogenously synthesized PAF and prostaglandins on the infection of mouse macrophages by *Letsbmanta (L.) amazonensis* was investigated, as well as the possible correlation between the effects of these inflammatory mediators with nitric oxide production. It was found that pretreatment of macrophages with 10^−5^ M of the PAF antagonists, BN-52021 or WEB-2086, increased macrophage infection by 17 and 59%, respectively. The cyclooxygenase inhibitor, indomethacin (10 *μ*g/ml), induced a significant inhibition which was reversed by addition of PGE (10-3 M) to the culture medium. These results suggested that the infection of macrophages by leisbmanla is inhibited by PAF and enhanced by prostaglandins and that these mediators are produced by macrophages during this infection. This was confirmed by addition of these mediators to the culture medium before infection; PAF (10^−6^, 10^−9^ and 10^−12^M) reduced significantly the infection whereas PGE_2_ (10^−5^ M) induced a marked enhancement. This effect of exogenous PAF on macrophage infection was reversed by the two PAF antagonists used in this study as well as by the inhibitor of nitric oxide synthesis, L-arginine methyl ester (100 mM). Taken together the data suggest that endogenous production of PAF and PGE_2_ exert opposing effects on *Lesbmana*–macrophage interaction and that nitric oxide may be involved in the augmented destruction of parasites induced by PAF.


Mediators of Inflammation 3, 137–141 (1994)

### Modulation of *Leishmania (L.) amazonensis* growth in cultured mouse macrophages by prostaglandins and platelet activating factor

M. V. C. Lonardoni,[1] C. L. Barbieri,[2] M. Russo[3] and S. Jancar[3,CA]

[1]Department of Biochemistry, Universidade Estadual de Maringá, Paraná; [2]Department of Microbiology, Immunology and Parasitology, Escola Paulista de Medicina, São Paulo; [3]Department of Immunology, Instituto de Ciências Biomédicas, Universidade de São Paulo, Av. Prof. Lineu Prestes 2415, CEP 05508-900, São Paulo, Brazil

[CA] Corresponding Author

**THE role of endogenously synthesized PAF and prostaglandins on the infection of mouse macrophages by *Leishmania (L.) amazonensis* was investigated, as well as the possible correlation between the effects of these inflammatory mediators with nitric oxide production. It was found that pretreatment of macrophages with $10^{-5}$ M of the PAF antagonists, BN-52021 or WEB-2086, increased macrophage infection by 17 and 59%, respectively. The cyclooxygenase inhibitor, indomethacin (10 μg/ml), induced a significant inhibition which was reversed by addition of $PGE_2$ ($10^{-5}$ M) to the culture medium. These results suggested that the infection of macrophages by *Leishmania* is inhibited by PAF and enhanced by prostaglandins and that these mediators are produced by macrophages during this infection. This was confirmed by addition of these mediators to the culture medium before infection; PAF ($10^{-6}$, $10^{-9}$ and $10^{-12}$ M) reduced significantly the infection whereas $PGE_2$ ($10^{-5}$ M) induced a marked enhancement. This effect of exogenous PAF on macrophage infection was reversed by the two PAF antagonists used in this study as well as by the inhibitor of nitric oxide synthesis, L-arginine methyl ester (100 mM). Taken together the data suggest that endogenous production of PAF and $PGE_2$ exert opposing effects on *Leishmania*–macrophage interaction and that nitric oxide may be involved in the augmented destruction of parasites induced by PAF.**

**Key words:** Leishmaniasis, *Leishmania (L.) amazonensis*, Macrophage infection, PAF, Prostaglandins

### Introduction

Species from the *Leishmania* genus are protozoans which present two forms during their life cycle, promastigotes which live in the digestive tract of the insect vector and amastigotes, obligate intracellular parasites which replicate in macrophages of their vertebrate hosts.

It has been shown that lymphokine activated mouse macrophages present an increased nitrite production which correlates with increased leishmanicidal activity of these cells.[1–3] It seems that production of nitric oxide (NO) is more important than superoxide in the intracellular parasite killing.[4] *Leishmania* destruction was shown to be completely reversed by structural analogues of L-arginine which inhibit NO production.[1–3] Conversely, parasite killing can proceed normally in a macrophage cell line deficient in the respiratory burst[4] and Kupffer cells which do not display an oxidative burst in response to *Leishmania*.[5] However, Andrade *et al.* showed that in the early phase of infection, a very low number of T cells is present at the site of inoculation of *Leishmania* in contrast to a massive influx of macrophages which rapidly take up the parasites.[6] Thus, it is unlikely that macrophage activation mediated by T cells would occur at this stage of infection. It is more plausible to think that macrophage responses induced by the parasite would influence the course of the disease. Indeed, Barcinski *et al.*[7] presented evidence that granulocyte–macrophage colony stimulating factor, a macrophage product, increases the infectivity of *Leishmania amazonensis* by protecting promastigotes from heat-induced death. A possible role for prostaglandins in the exacerbation of the disease in *L. major*-infected BALB/c mice was suggested by Farrell and Kirkipatrick.[8] It has been shown that an increased production of $PGE_2$, $PGF_{2\alpha}$, $LTC_4$, $TXB_2$ and $PGD_2$ occurs during the course of murine infection with *L. donovani*.[9,10]

The lipid inflammatory mediators derived from arachidonic acid metabolism (eicosanoids) and PAF (platelet activating factor) may exert important autocrine effects on macrophages. Prostaglandins of the E series increase cAMP[11–13] and by this mechanism they modulate several macrophage functions.[14] PAF is an important amplifier of biological processes in several cell types. In macrophages, PAF inhibits expression of class II molecules,[15] modulates IL-1 production in LPS-stimulated monocytes[16] and stimulates TNF production.[17] To date, PAF involvement in *Leishmania* infection has not been reported.

© 1994 Rapid Communications of Oxford Ltd

The purpose of the present study was to investigate the role of endogenously produced prostaglandins and PAF in *in vitro* infection of mouse macrophages with *L. (L.) amazonensis*. Macrophages of BALB/c mice were infected with *L. (L.) amazonensis* amastigotes and the phagocytic index was determined in presence and absence of PAF antagonists, WEB-2086 or BN-52021, and an inhibitor of prostaglandin synthesis, indomethacin. The effect of PAF and $PGE_2$ added to the culture medium was also investigated. The possible involvement of NO was studied by using L-arginine methyl ester (L-NAME) and an inhibitor of NO synthesis.

### Materials and Methods

*Animals:* BALB/c mice (20–30 g) and outbred Golden hamsters 2–3 months old from our own animal facilities were used.

*Isolation of* L. (L.) amazonensis *amastigotes from hamster foot pads: L. (L.) amazonensis* amastigotes ($10^7$ parasites/ml) were transferred every 4 to 6 weeks to hamsters by inoculation into foot pads. Amastigote suspensions were prepared by homogenization of excised lesions in RPMI 1640 medium containing 10% foetal calf serum (FCS) with a Poter glass homogenizer and the parasites separated by centrifugation at 1 400 × ***g*** for 10 min.

*Macrophage cultures and* L. (L.) amazonensis *infection:* Macrophages were collected in phosphate buffered saline (PBS) from peritoneal cavities of BALB/c mice. About $4 \times 10^5$ cells were allowed to attach to round 13 mm glass coverslips. The non-adherent cells were removed by rinsing the coverslips with PBS. Coverslips were placed in 16 mm diameter wells of Costar plates containing 0.5 ml of RPMI 1640 plus 10% FCS, 100 U of penicillin and 100 μg of streptomycin per ml and kept in a 5% $CO_2$ humid atmosphere at 37°C. After 24 h, *L. (L.) amazonensis* amastigotes were added to the macrophage monolayers at a cell ratio of 3 parasites/macrophage at 37°C. At different times after infection, the coverslips were washed with PBS, the cells fixed in absolute methanol for 10 min and stained with Giemsa, dried, mounted on glass slides and examined microscopically.

Results were expressed by phagocytic index which is the product of the percentage of infected macrophages times the average number of amastigotes per macrophage.

*Evaluation of nitric oxide production:* Nitrite is an oxidation product formed by nitric oxide in an aqueous solution. The nitrite concentration was measured in the culture supernatant media by the method previously described by Green *et al.*[2] Briefly, 50 μl aliquots of samples in triplicate were added to 50 μl of Griess reagent in 96-well flat-bottomed plates. The absorbance was read at 550 nm (Dynatech MR5000) after 10 min of reaction and $NO_2^-$ concentration was deduced from a standard curve using concentrations from 1 to 5 μM of sodium nitrite in culture media.

*Drug treatments:* Indomethacin (10 μg/ml) prepared in Tris–HCl (1 M, pH 8.0) was added to the macrophage culture medium 24 h before infection. The PAF antagonists, WEB-2086 and BN-52021, were added to the culture medium 1 h before infection and every 12 h thereafter at concentrations ranging from $10^{-5}$ to $10^{-7}$ M. The diluent for BN-52021 was provided by the drug company and WEB-2086 was dissolved in PBS. The agonists PAF ($10^{-6}$, $10^{-9}$ and $10^{-12}$ M) and $PGE_2$ ($10^{-5}$ M) were added to the culture medium at the moment of infection. PAF and $PGE_2$ were supplied in ethanol and were further diluted in saline containing 0.25% BSA and PBS, respectively. The concentration of ethanol in the culture medium never exceeded 0.1%. The effect of all diluents used was assayed. At the concentrations used, the drugs did not significantly affect macrophage viability as assessed by the Trypan blue exclusion test.

*Drugs and reagents:* Cell culture medium (RPMI 1640), indomethacin, L-glutamine, penicillin, streptomycin and N-omega-nitro-L-arginine methyl ester hydrochloride (L-NAME) were all purchased from Sigma Chem. Co. (USA); $PGE_2$ from UpJohn Co. (USA) and PAF from Bachem (Switzerland). BN-52021 and WEB-2086 were kindly supplied by Institut Henri Beaufour (France) and Boehringer–Ingelheim (Germany), respectively.

*Statistical analysis:* Student's *t*-test for paired samples and analysis of variance were used to evaluate the significance of the data ($p < 0.01$).

### Results

*Effect of endogenous PAF and prostaglandins on* L. (L.) amazonensis *infected macrophages:* In order to investigate the role of endogenously generated PAF and prostaglandins in *L. (L.) amazonensis* macrophage infection, the macrophages were treated with either the PAF antagonists (BN-52021 or WEB-2086) or the cyclooxygenase inhibitor (indomethacin) before infection, and the phagocytic index was determined 48 h later. The two antagonists were added to the culture medium 1 h before infection and every 12 h thereafter while indomethacin was added 24 h before infection. The results obtained (Fig. 1) show that pre-treatment of macrophages with BN-52021 or WEB-2086 significantly increased the macrophage infection (17.3 and 59.4% increase, respectively). Indomethacin, however, induced a significant inhibition. Addition of $PGE_2$ ($10^{-5}$ M) to indomethacin-treated macrophages reversed this inhibition.

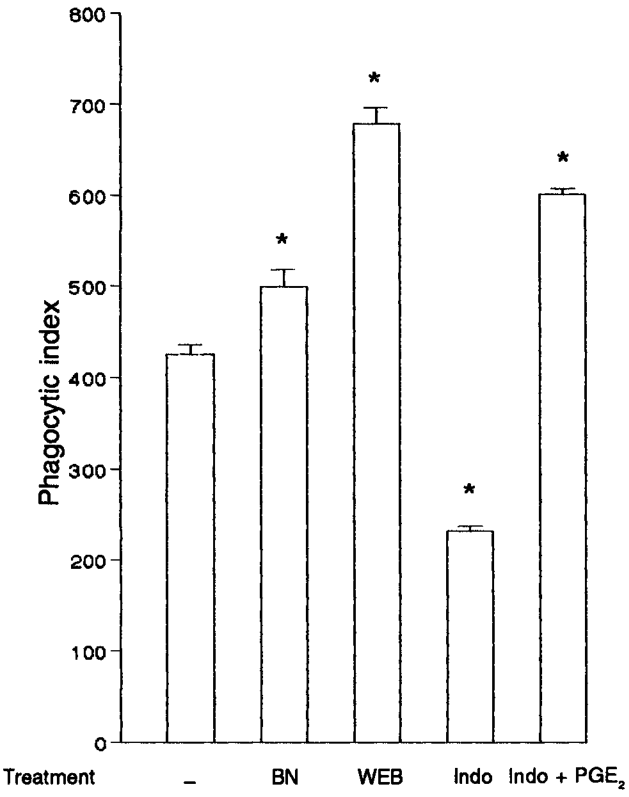

FIG. 1. Effect of endogenous PAF and prostaglandins on *L. (L.) amazonensis* infected macrophages. Mouse peritoneal macrophages treated with indomethacin (10 μg/ml), BN-52021 ($10^{-5}$M), WEB-2086 ($10^{-5}$M) and indomethacin + $PGE_2$ ($10^{-6}$M) before infection with *L. (L.) amazonensis* amastigotes. Indomethacin and the PAF antagonists were added to the culture medium 24 and 1 h before infection, respectively. The phagocytic index was determined 48 h later. The controls represent the values obtained in non-treated macrophages. The results were obtained by counting at least 200 macrophages per duplicate coverslip. Error bars show standard deviations of three experiments (*$p < 0.01$ compared with the non-treated group).

Table 1 shows that WEB-2086 increased the infection dose dependently, a maximum potentiation being achieved with $10^{-5}$M, although at $10^{-6}$ and $10^{-7}$M the drug was also significantly effective. The control group treated with the diluents of the drugs did not differ from the non-treated group.

*Effect of exogenous PAF and $PGE_2$ on* L. (L.) amazonensis *infected macrophages:* The results obtained with the PAF antagonists and indomethacin suggest that PAF and prostaglandins endogenously generated modulate the *in vitro* infection. In order to evaluate whether addition of PAF or $PGE_2$ to the culture medium influence the infection, these mediators were added to the culture medium at the moment of infection and the phagocytic index determined 48 h later. The results obtained show that PAF ($10^{-6}$, $10^{-9}$ and $10^{-12}$M) reduced the infection dose dependently whereas $PGE_2$ ($10^{-5}$M) induced a marked enhancement (Fig. 2). In some experiments PAF was added to culture medium 24 h before infection. In this case it caused 26.5 and 74.6% of inhibi-

**Table 1.** Effect of WEB-2086 on the infection of macrophages by *L. (L) amazonensis*

| Concentration of WEB-2086 (M) | Time after infection (h) 24 | 48 | 72 |
|---|---|---|---|
| Control | 249.31 ± 9.03 | 408.91 ± 5.34 | 658.08 ± 9.83 |
| $10^{-7}$ | 388.83 ± 7.27* (55.96) | 505.06 ± 21.80* (23.51) | 563.71 ± 11.85* (14.34) |
| $10^{-6}$ | 412.97 ± 5.80* (57.95) | 577.30 ± 7.18* (36.08) | 722.26 ± 8.80* (18.62) |
| $10^{-5}$ | 492.43 ± 14.71* (88.34) | 696.72 ± 16.51* (64.23) | 850.87 ± 17.90* (39.76) |

Phagocytic index obtained in macrophages infected with *L. (L) amazonensis* in the absence (Control) or presence of WEB-2086, added to the culture medium 1 h before infection. Data represent the mean ± S.E.M. of three experiments in duplicate. *$p < 0.01$ compared with the group treated with the diluent. Data in brackets represent the percentage of enhancement.

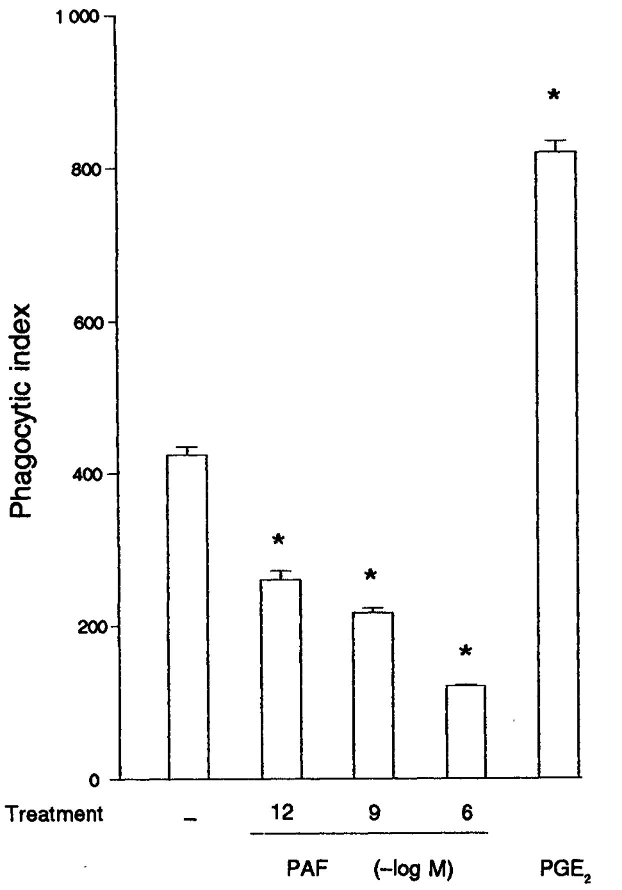

FIG. 2. Effect of exogenous PAF and $PGE_2$ on *L. (L.) amazonensis* infected macrophages. Mouse peritoneal macrophages treated with PAF ($10^{-12}$, $10^{-9}$ and $10^{-6}$ M) and $PGE_2$ ($10^{-5}$ M) at the moment of infection with *L. (L.) amazonensis* amastigotes and phagocytic index determined 48 h later. Non-treated macrophages were used as control. The results were obtained by counting at least 200 macrophages per duplicate coverslip. Error bars show standard deviations of six experiments (*$p < 0.01$ compared with the non-treated group).

**Table 2.** Effect of PAF antagonists on the PAF induced inhibition of macrophages infection by *L. (L) amazonensis* amastigotes

| Group | Treatment[a] | Agonist[b] ($10^{-6}$ M) | Phagocytic index[c] |
|---|---|---|---|
| I | — | — | 425.57 ± 10.26 |
| II | — | PAF | 125.17 ± 2.36* |
| III | BN-52021 ($10^{-5}$ M) | PAF | 223.88 ± 7.76** |
| IV | WEB-2086 ($10^{-5}$ M) | PAF | 181.95 ± 4.44** |

[a]The antagonists were given 1 h before infection.
[b]PAF was added at the moment of infection.
[c]The phagocytic index was determined 48 h after infection of macrophages with *L. (L.) amazonensis*.
Data represent the mean ± S.E.M. of three experiments in duplicate
*$p < 0.01$ compared with group I.
**$p < 0.01$ compared with group II.

tion at $10^{-9}$ and $10^{-6}$ M, respectively. At $10^{-12}$ M it was no longer effective. The control groups treated with diluents of PAF and $PGE_2$ did not differ from the non-treated group.

*Effect of PAF antagonists on PAF induced inhibition of* L. (L.) amazonensis *macrophage infection:* The ability of PAF antagonists to inhibit the PAF induced effect was investigated. Macrophages were treated with $10^{-5}$ M of the antagonists BN-52021 or WEB-2086, 1 h before addition of PAF at $10^{-6}$ M concentration to the culture medium and the phagocytic index was determined 48 h later. Results presented in Table 2 confirmed that PAF induced a marked inhibition of the phagocytic index and showed that both PAF antagonists were able to significantly reverse this effect.

*Effect of L-NAME on PAF induced inhibition of* L. (L.) amazonensis *macrophage infection:* The effect of L-NAME was studied in combination with PAF. The macrophages were treated with L-NAME, 1 h before addition of PAF ($10^{-6}$ M) and parasites to the culture medium and the phagocytic index was determined 48 h later. Controls were carried out by treating the macrophages with either L-NAME or PAF alone. Fig. 3 shows that the inhibitory effect of PAF on the *L. (L.) amazonensis* macrophage infection was reversed by 100 mM L-NAME. This figure also shows that L-NAME increased the phagocytic index of macrophages treated with or without PAF. In addition, we assayed the NO release in the supernatants. The concentration of nitrites/nitrates in these supernatants was below the detection limit allowed by the assay employed (data not shown). The efficacy of L-NAME to inhibit NO production was tested on macrophages from BCG infected mice. It was found that BCG-activated macrophages released 0.929 ± 0.054 nmol of NO/$10^5$ peritoneal cells after 48 h in culture medium and that pre-treatment of macrophages with 100 mM of L-NAME significantly inhibited this production (0.402 ± 0.018 nmol/$10^5$ peritoneal cells).

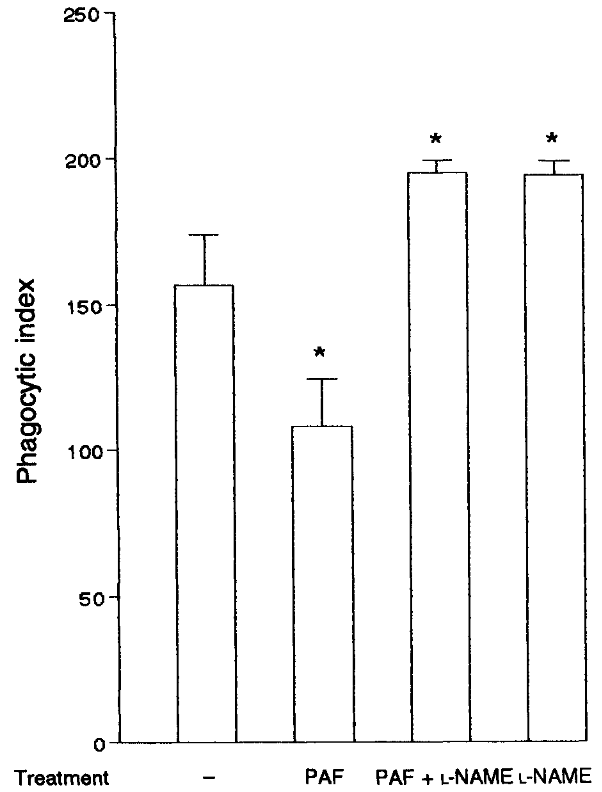

FIG. 3. Effect of L-NAME on PAF induced inhibition of *L. (L.) amazonensis* macrophage infection. Mouse peritoneal macrophages pretreated for 1 h with L-NAME (100 μM) followed by administration of PAF ($10^{-6}$ M) at the moment of infection with *L. (L.) amazonensis* amastigotes. Phagocytic index was determined 48 h later. The results were obtained by counting at least 200 macrophages per triplicate coverslip. Error bars show standard deviations of three experiments (*$p < 0.01$ compared with the non-treated group).

### Discussion

In leishmaniasis, as in other intracellular parasite infections, macrophages are both the host and the effector cell. Several studies focused on the influence of T cells and their products on macrophages activation which would lead either to healing or exacerbation of the disease (for review see Locksley and Scott[18]). However, few studies have analysed the consequences of the initial interaction between macrophage and parasites on parasite multiplication.

In the present work we showed that treatment of macrophages with indomethacin, an inhibitor of prostaglandins synthesis, before infection with *L. (L.) amazonensis* significantly inhibited parasite growth. This result indicates that prostaglandins are released by macrophages during the infection and that they favour parasite survival possibly by inhibiting activation of the macrophage. Since the effect of indomethacin was reversed by the addition of $PGE_2$ to the culture medium, it is likely that this cyclooxygenase metabolite is the one responsible for the observed effect. Our findings are in agreement with those reported by Buchmüller-Rouiller *et al.*[19]

showing that $PGE_2$ inhibits the leishmanicidal activity of macrophages activated with ionophore and LPS.

That macrophages are able to produce prostaglandins when infected with *Leishmania* has been shown by studies *in vitro* and *in vivo*.[9,10] Prostaglandin $E_2$ is known to exert negative feedback on macrophage activation,[20] an effect probably mediated by an increased intracellular level of cAMP.[19] In our model, it is probable that prostaglandins generated by the infected macrophages inhibited their capacity to control parasite multiplication. In support of this assumption it has been shown that treatment of susceptible BALB/c mice infected with *L. major* with indomethacin significantly inhibited the number of metastatic lesions.[8] However, this effect might not rely entirely on inhibition of macrophage function since prostaglandins also inhibit lymphocyte proliferation induced by Con A and parasite antigens.[8]

To study the role of PAF on this infection, two specific PAF antagonist, BN-52021[21] or WEB-2086[22] were added to the culture medium before infection with *L. (L.) amazonensis*. These treatments increased the phagocytic index indicating that PAF is generated endogenously by infected macrophages and in turn it exerts a marked inhibition of parasite growth. This was confirmed by experiments where PAF was added exogenously to culture medium of infected macrophages. In this case, a clear dose dependent inhibition of parasite multiplication was observed. We also showed that the doses of the PAF antagonists used in this study were effective to antagonize PAF receptors in murine macrophages since both antagonists were able to reverse the inhibitory effect of PAF.

It is known that macrophages can generate PAF when properly stimulated;[17] however, to our knowledge this is the first report to show that PAF exerts such a relevant effect on leishmanial infection.

Another substance that is recognized as a potent leishmanicidal agent *in vivo* and *in vitro* is nitric oxide (for review see Liew and Cox[23]). Thus, we reasoned whether the inhibitory effect of PAF on leishmanial growth could be mediated by NO. To this end we added an inhibitor of NO synthesis (L-NAME) to infected macrophages in the presence or absence of PAF. In both situations L-NAME increased parasite multiplication. These results, although indirect, strongly suggest the participation of NO as the active molecule that mediates the PAF induced macrophage leishmanicidal activity.

The present work showed that $PGE_2$ has a stimulatory effect whereas PAF has an inhibitory effect on *L. (L.) amazonensis* infection in mouse macrophages. It also showed that the leishmanicidal effect of PAF is mediated by NO. It will be of interest to measure the levels of $PGE_2$ and PAF released by infected macrophages obtained from resistant and susceptible strains of mouse and also to determine the effect of these lipid mediators on the course of *in vivo* infection.

### References

1. Liew FY, Millott S, Parkinson C, Palmer RMJ, Moncada S. Macrophage killing of *Leishmania* parasite *in vivo* is mediated by nitric oxide from L-arginine. *J Immunol* 1990; **144**: 4794–4797.
2. Green SJ, Meltzer MS, Hibbs Jr JB, Nacy CA. Activated macrophages destroy intracellular *Leishmania major* amastigotes by an L-arginine-dependent killing mechanism. *J Immunol* 1990; **144**: 278–283.
3. Mauël J, Ransijn A, Buchmüller-Rouiller Y. Killing of *Leishmania* parasites in activated murine macrophages is based on an L-arginine-dependent process that produces nitrogen derivatives. *J Leuk Biol* 1991; **49**: 73–82.
4. Scott P, James S, Sher A. The respiratory burst is not required for killing intracellular and extracellular parasites by a lymphokine-activated macrophage cell line. *Eur J Immunol* 1985; **15**: 553–558.
5. Crocker PR, Davis EV, Blackwell JM. Variable expression of the murine natural resistance gene *Lsh* in different macrophage populations infected *in vitro* with *Leishmania donovani*. *Parasite Immunol* 1987; **9**: 705–719.
6. Andrade ZA, Reed SG, Roters SB, Sadigursky M. Immunopathology of experimental cutaneous leishmaniasis. *Am J Pathol* 1984; **114**: 137–148.
7. Barcinski MA, Schechtman D, Quinta, *et al.* Granulocyte-macrophage colony-stimulating factor increases the infectivity of *Leishmania amazonensis* by protecting promastigotes from heat-induced death. *Infect Immun* 1992; **60**: 3523–3527.
8. Farrell JP, Kirkipatrick CE. Experimental cutaneous leishmaniasis. II. A possible role for prostaglandins in exacerbation of disease in *Leishmania major*-infected BALB/c mice. *J Immunol* 1987; **138**: 902–907.
9. Reiner NE, Malemud CJ. Arachidonic acid metabolism in murine Leishmaniasis (*donovani*): *ex-vivo* evidence for increased cyclooxygenase and 5-lipoxygenase activity in spleen cells. *Cell Immunol* 1984; **88**: 501–510.
10. Reiner NE, Malemud CJ. Arachidonic acid metabolism by murine peritoneal macrophages infected with *Leishmania donovani*: *in vitro* evidence for parasite-induced alterations in cyclooxygenase and lipoxygenase pathways. *J Immunol* 1985; **134**: 556–563.
11. Goodwin JS, Ceuppens J. Regulation of the immune response by prostaglandins. *J Immunol* 1983; **3**: 295–297.
12. Rincon M, Tugores A, Lopez-Rivas A, *et al.* Prostaglandin E and the increase of intracellular cAMP inhibit the expression of interleukin-2 receptors in human T cells. *Eur J Immunol* 1988; **18**: 1791–1794.
13. Papadogiannakis N, Nordstrom TE, Anderson LC, Wolff CHJ. cAMP inhibits the OKT3-induced increase in cytoplasmatic free calcium in the Jukart T cell line. *Eur J Immunol* 1989; **19**: 1953–1958.
14. Minakuchi R, Wacholtz MC, Davis LS, Lipsky PE. Delineation of the mechanism of inhibition of human T-cell activation by $PGE_2$. *J Immunol* 1990; **145**: 2616–2625.
15. Gehard BM, Bazan HEP, Bazan NG. BN 52021 blocks PAF-mediated suppression of cellular immunity. In: Braquet P, ed. *Ginkgolides—chemistry, biology, pharmacology and clinical perspectives*. Barcelona: Prous. Sci. Publish., 1988; 703–729.
16. Pignol B, Henane S, Mencia-Huerta JM, Rola-Pleszczynski M, Braquet P. Effect of PAF-acether and its specific antagonist, BS 52021, on interleukin-1 synthesis and release by rat monocytes. *Prostaglandins* 1987; **33**: 391–397.
17. Poubelle PE, Gingras D, Demers, *et al.* Platelet-activating factor (PAF-acether) enhances the concomitant production of tumor necrosis factor-alpha and IL-1 by subsets of human monocytes. *J Immunol* 1991; **72**: 181–187.
18. Locksley RM, Scott P. Helper T-cell subsets in mouse leishmaniasis: induction, expansion and effector functions. *Immunol Today* 1991; **12**: A58–A61.
19. Buchmüller-Rouiller Y, Betz-Corradini S, Mauël J. Differential effects of prostaglandins on macrophage activation induced by calcium ionophore or IFN-gamma. *J Immunol* 1992; **148**: 1171–1175.
20. Schultz RM. Factors limiting tumoricidal functions of interferon-induced effector systems. *Cancer Immunol Immunother* 1978; **10**: 61–66.
21. Braquet P, Godfroid JJ. PAF-acether specific binding sites. Design of specific antagonists. *Trends Pharmacol Sci* 1986; **7**: 397–403.
22. Casals-Stenzel J, Muacevic R, Weber H. Pharmacological actions of WEB 2086, a new specific antagonist of PAF. *J Pharmac Exp Therap* 1987; **241**: 947–981.
23. Liew FY, Cox FEG. Nonspecific defence mechanism: the role of nitric oxide. *Immunol Today* 1991; **12**: A17–A21.

ACKNOWLEDGEMENTS. The authors thank Maria de Fátima Farias P. Carvalho and Dunia Rodriguez for technical assistance. This work was supported by Fundação de Amparo à Pesquisa do Estado de São Paulo (FAPESP) and Conselho Nacional de Desenvolvimento Científico e Tecnológico (CNPq) from Brazil.

**Received 22 November 1993; accepted in revised form 3 January 1994**

## References

[PDF_00473] Andrade Z. A., Reed S. G., Roters S. B., Sadigursky M. (1984). Immunopathology of experimental cutaneous leishmaniasis.. Am J Pathol.

[PDF_00475] Barcinski M. A., Schechtman D., Quintao L. G., Costa D. de A., Soares L. R., Moreira M. E., Charlab R. (1992). Granulocyte-macrophage colony-stimulating factor increases the infectivity of Leishmania amazonensis by protecting promastigotes from heat-induced death.. Infect Immun.

[PDF_00509] Buchmüller-Rouiller Y., Betz-Corradin S., Mauël J. (1992). Differential effects of prostaglandins on macrophage activation induced by calcium ionophore A23187 or IFN-gamma.. J Immunol.

[PDF_00516] Casals-Stenzel J., Muacevic G., Weber K. H. (1987). Pharmacological actions of WEB 2086, a new specific antagonist of platelet activating factor.. J Pharmacol Exp Ther.

[PDF_00501] Chung H., Fried J., Jarabak J. (1987). Irreversible inhibition of the human placental NADP-linked 15-hydroxyprostaglandin dehydrogenase/9-ketoprostaglandin reductase by glutathione thiosulfonate.. Prostaglandins.

[PDF_00470] Crocker P. R., Davies E. V., Blackwell J. M. (1987). Variable expression of the murine natural resistance gene Lsh in different macrophage populations infected in vitro with Leishmania donovani.. Parasite Immunol.

[PDF_00478] Farrell J. P., Kirkpatrick C. E. (1987). Experimental cutaneous leishmaniasis. II. A possible role for prostaglandins in exacerbation of disease in Leishmania major-infected BALB/c mice.. J Immunol.

[PDF_00488] Goodwin J. S., Ceuppens J. (1983). Regulation of the immune response by prostaglandins.. J Clin Immunol.

[PDF_00461] Green S. J., Meltzer M. S., Hibbs J. B., Nacy C. A. (1990). Activated macrophages destroy intracellular Leishmania major amastigotes by an L-arginine-dependent killing mechanism.. J Immunol.

[PDF_00518] Liew F. Y., Cox F. E. (1991). Nonspecific defence mechanism: the role of nitric oxide.. Immunol Today.

[PDF_00458] Liew F. Y., Millott S., Parkinson C., Palmer R. M., Moncada S. (1990). Macrophage killing of Leishmania parasite in vivo is mediated by nitric oxide from L-arginine.. J Immunol.

[PDF_00507] Locksley R. M., Scott P. (1991). Helper T-cell subsets in mouse leishmaniasis: induction, expansion and effector function.. Immunol Today.

[PDF_00464] Mauël J., Ransijn A., Buchmüller-Rouiller Y. (1991). Killing of Leishmania parasites in activated murine macrophages is based on an L-arginine-dependent process that produces nitrogen derivatives.. J Leukoc Biol.

[PDF_00496] Minakuchi R., Wacholtz M. C., Davis L. S., Lipsky P. E. (1990). Delineation of the mechanism of inhibition of human T cell activation by PGE2.. J Immunol.

[PDF_00493] Papadogiannakis N., Nordström T. E., Andersson L. C., Wolff C. H. (1989). cAMP inhibits the OKT3-induced increase in cytoplasmic free calcium in the Jurkat T cell line: the degree of inhibition correlates inversely with the amount of CD3 binding ligand used.. Eur J Immunol.

[PDF_00504] Poubelle P. E., Gingras D., Demers C., Dubois C., Harbour D., Grassi J., Rola-Pleszczynski M. (1991). Platelet-activating factor (PAF-acether) enhances the concomitant production of tumour necrosis factor-alpha and interleukin-1 by subsets of human monocytes.. Immunology.

[PDF_00484] Reiner N. E., Malemud C. J. (1985). Arachidonic acid metabolism by murine peritoneal macrophages infected with Leishmania donovani: in vitro evidence for parasite-induced alterations in cyclooxygenase and lipoxygenase pathways.. J Immunol.

[PDF_00481] Reiner N. E., Malemud C. J. (1984). Arachidonic acid metabolism in murine leishmaniasis (Donovani): ex-vivo evidence for increased cyclooxygenase and 5-lipoxygenase activity in spleen cells.. Cell Immunol.

[PDF_00490] Rincón M., Tugores A., López-Rivas A., Silva A., Alonso M., De Landázuri M. O., López-Botet M. (1988). Prostaglandin E2 and the increase of intracellular cAMP inhibit the expression of interleukin 2 receptors in human T cells.. Eur J Immunol.

[PDF_00467] Scott P., James S., Sher A. (1985). The respiratory burst is not required for killing of intracellular and extracellular parasites by a lymphokine-activated macrophage cell line.. Eur J Immunol.

